# Prevalence and incidence of intraventricular conduction disturbances among Chinese adults: Results from the Kailuan study

**DOI:** 10.3389/fcvm.2022.959781

**Published:** 2022-09-20

**Authors:** Binhao Wang, Zhao Wang, Xiaolei Yang, Xu Han, Yiheng Yang, Huimin Chu, Shouling Wu, Yunlong Xia

**Affiliations:** ^1^Department of Cardiology, The First Affiliated Hospital of Dalian Medical University, Dalian, China; ^2^Arrhythmia Center, Ningbo First Hospital, Ningbo, China; ^3^Department of Ultrasonography, Ningbo First Hospital, Ningbo, China; ^4^Department of Cardiology, Kailuan General Hospital, Tangshan, China

**Keywords:** intraventricular conduction disturbances, prevalence, incidence, electrocardiography, cardiovascular diseases

## Abstract

**Objectives:**

Epidemiological data on intraventricular conduction disturbances (IVCDs) are limited in the Chinese population. We aimed to investigate the prevalence and incidence of different types of IVCDs among Chinese adults.

**Methods:**

From June 2006 to October 2007, a total of 100,250 individuals from the Kailuan Study underwent electrocardiogram examinations. Follow-up visits were performed biennially. The prevalence and incidence of right bundle branch block (RBBB), incomplete RBBB (IRBBB), left bundle branch block (LBBB), incomplete LBBB, left anterior fascicular block (LAFB), left posterior fascicular block (LPFB) and non-specific IVCD (NIVCD) were calculated. Their association with risk factors and cardiovascular diseases was also investigated.

**Results:**

The prevalence and incidence of all IVCDs were 3.19 and 1.70%, respectively. RBBB, IRBBB, and LAFB were the IVCD types that had the highest prevalence and incidence. The prevalence (3.67% vs. 1.29%; *P* < 0.001) and incidence (1.93% vs. 0.84%; *P* < 0.001) of IVCDs were higher among males than among females. The prevalence and incidence of most IVCD types increased with age. IRBBB, LBBB, and LAFB were significantly associated with hypertension. LBBB and NIVCD were associated with diabetes mellitus. In addition, LBBB and LAFB were related to prior myocardial infarction.

**Conclusion:**

IVCDs differ in prevalence and incidence according to sex and age group. They also show disparate associations with other cardiovascular comorbidities. These differences need to be considered in daily clinical practice.

## Introduction

Electrocardiography (ECG) has become one of the most commonly used diagnostic tools performed in routine clinical settings. As a result, intraventricular conduction disturbances (IVCDs) have been identified more frequently, raising questions, and often concerns. Most of the investigations regarding IVCDs were performed among subjects with high risks (e.g., heart failure and myocardial infarction) (1, 2). The majority of studies on the general population have focused on certain types of IVCDs, such as left bundle branch (LBBB) and right bundle branch block (RBBB) (3, 4). Several studies have provided data about the prevalence of all types of IVCDs in the general population. Haataja et al. (5) performed an epidemiological study of IVCDs in the general Finnish population. Monin et al. investigated the prevalence of IVCDs in a large French population (6). They both found that IVCDs differed in prevalence between sexes and age groups. However, studies in Asian populations are scarce, especially in the Chinese population. In addition, little is known about the incidence of IVCDs in the general population.

The Kailuan Study is a prospective cohort study conducted in the community of Kailuan in Tangshan, Hebei Province, China (7). Consistent follow-up and complete data records provide us with the opportunity to determine the prevalence and incidence of IVCDs and their association with some major cardiovascular diseases (CVDs) in a Chinese community-based population.

## Materials and methods

### Study design and population

This study was conducted based on data from the Kailuan Study. From June 2006 to October 2007, a total of 101,510 participants (aged 18–98, 81,110 men and 20,400 women) were recruited to participate in the Kailuan Study (1st examination). Follow-up visits were performed every 2 years. The objective of the present study was to investigate the prevalence and incidence of IVCDs; therefore, Kailuan participants without ECG recordings were excluded. Finally, 100,250 individuals (80,056 men and 20,194 women) were included in this study at baseline. Follow-up examinations were performed every 2 years (2nd examination, 2008–2009; 3rd examination, 2010–2011; 4th examination, 2012–2013).

### Definition of intraventricular conduction disturbances

A 10-s resting 12-lead ECG was recorded for every participant between 6:00 a.m. and 9:00 a.m. (before breakfast) after participants had been comfortably resting in the supine position for 5 min in a quiet room. Manual ECG analysis was performed by two independent investigators. Seven types of IVCDs were classified according to the American Heart Association (AHA)/American College of Cardiology Foundation (ACCF)/Heart Rhythm Society (HRS) recommendations (8): RBBB, incomplete RBBB (IRBBB), LBBB, incomplete LBBB (ILBBB), non-specific IVCD (NIVCD), left anterior fascicular block (LAFB), and left posterior fascicular block (LPFB).

RBBB was defined by (1) a QRS duration greater than or equal to 120 ms; (2) rsr’, rsR’, or rSR’ in leads V_1_ or V_2_ and the R’ or r’ deflection usually wider than the initial R wave; (3) an S wave of greater duration than the R wave or greater than 40 ms in leads I and V_6_; and (4) pure dominant R waves in V_1_ accompanied by normal R peak time in leads V_5_ or V_6_ with an R peak time greater than 50 ms in lead V_1_. IRBBB was defined by a QRS duration between 110 and 119 ms; the other criteria remained the same as those for RBBB. LBBB was defined by (1) a QRS duration greater than or equal to 120 ms; (2) a broad notched or slurred R wave in leads I, aVL, V_5_, and V_6_ and an occasional RS pattern in V_5_ and V_6_ attributed to displaced transition of the QRS complex; (3) absent q waves in leads I, V_5_, and V_6_ (a narrow q wave may be present in lead aVL); (4) an R peak time greater than 60 ms in leads V_5_ and V_6_ but normal in leads V_1_, V_2_, and V_3_ with discernable small initial r waves in the above leads; and (5) possible changes in the QRS axis in the frontal plane, the ST segment, and the T wave. ILBBB was defined by (1) a QRS duration between 110 and 119 ms; (2) presence of left ventricular hypertrophy pattern; (3) an R peak time greater than 60 ms in leads V_4_, V_5_, and V_6_; and (4) the absence of a q wave in leads I, V_5_, and V_6_. NIVCD was defined by a QRS duration greater than 110 ms without the criteria for RBBB and LBBB. LAFB was defined by (1) a frontal plane axis between -45° and -90°; (2) a qR pattern in lead aVL; (3) an R peak time in lead aVL greater than 45 ms; and (4) a QRS duration less than 120 ms. LPHB was defined by (1) a frontal plane axis between 90° and 180°; (2) an rS pattern in leads I and aVL; (3) a qR pattern in leads III and aVF; and (4) a QRS duration less than 120 ms.

### Collection and definitions of potential covariates and cardiovascular diseases

A questionnaire that included health-related lifestyle factors, disease history, and use of antihypertensive and antidiabetic medications was conducted during the visits. Body mass index was calculated as the weight (kg) divided by the height squared (m^2^). Resting heart rate was obtained by ECG. Smoking status was grouped into three categories: current smoker, former smoker, and never smoker. Similarly, drinking status was also divided into three categories: current drinker, former drinker, and never drinker. Blood pressure was measured while seated in an upright position after 5 min. Fasting (> 8 h) blood samples were collected and processed for analysis, and fasting plasma glucose was measured. Hypertension (HTN) was defined as a systolic blood pressure ≥ 140 mmHg and/or diastolic blood pressure ≥ 90 mmHg or a self-reported history of HTN with current antihypertensive medication use. Diabetes mellitus (DM) was defined as fasting plasma glucose ≥ 7.0 mmol/L, random plasma glucose ≥ 11.1 mmol/L or a self-reported history of DM with current use of antidiabetic medication. A self-reported history of myocardial infarction (MI) and stroke was recorded and further ascertained by linking to the Medical Insurance Center of Tangshan City.

### Statistical analysis

Baseline characteristics were compared between participants with and without IVCDs. Normally distributed continuous variables are expressed as means ± standard deviations, and medians (interquartile ranges) are used for variables with a skewed distribution. Categorical variables are expressed as absolute numbers (percentages). Continuous variables were compared using the *t*-test and Mann-Whitney U test for normally and non-normally distributed data, respectively. Categorical variables were compared using the chi-square test or Fisher’s exact test where appropriate.

The results are shown as percentages for the prevalence and incidence proportion (IP) and cases per 1,000 person-years for incidence density (ID). The prevalence and incidence in each group were compared by binary logistic regression adjusted for age or sex. The association between IVCDs and CVDs was evaluated by logistic regression. According to a rule of thumb, logistic models should be used with a minimum of 10 events per predictor (9). Therefore, an age- and sex-adjusted logistic model was run for the types of IVCDs that did not have enough cases for the multivariate adjusted model. For the multivariate adjusted logistic model, demographic data, lifestyle factors, medications and CVDs were included. Odds ratios (ORs) and 95% confidence intervals (CIs) were calculated. All analyses were conducted with SAS 9.3 (SAS Institute, Cary, NC). A two-tailed *P*-value < 0.05 was considered statistically significant.

## Results

### Baseline characteristics

A total of 3,201 participants had IVCDs at baseline, and the prevalence of all IVCDs was 3.19%. The individuals with IVCDs were older. The percentages of males, hypertension, diabetes mellitus, prior myocardial infarction, and prior stroke were higher among the subjects with IVCDs (Table 1).

**TABLE 1:** Baseline characteristics of the study population.

	No IVCDs (*n* = 97,049)	With IVCDs (*n* = 3,201)	*P*-value
Age, years	51.7 ± 12.6	59.7 ± 13.3	<0.001
Male, *n* (%)	77,116 (79.5)	2,940 (91.8)	<0.001
Body mass index, kg/m^2^	25.0 ± 3.5	25.0 ± 3.5	0.820
Current smoker, *n* (%)	32,511 (33.5)	1,104 (34.5)	0.243
Current drinker, *n* (%)	35,229 (36.3)	1,175 (36.7)	0.638
Hypertension, *n* (%)	42,799 (44.1)	1,684 (52.6)	<0.001
Antihypertensive medication, *n* (%)	10,772 (11.1)	531 (16.6)	<0.001
Diabetes mellitus, *n* (%)	9,123 (9.4)	360 (11.2)	<0.001
Antidiabetic medication, *n* (%)	2,329 (2.4)	122 (3.8)	<0.001
Prior myocardial infarction, *n* (%)	1,262 (1.3)	92 (2.9)	<0.001
Prior stroke, *n* (%)	2,426 (2.5)	162 (5.1)	<0.001
Systolic blood pressure, mmHg	130.9 ± 21.0	135.6 ± 21.9	<0.001
Diastolic blood pressure, mmHg	83.5 ± 11.8	84.5 ± 12.0	<0.001
Resting heart rate, beats per minute	73.9 ± 10.3	73.2 ± 11.4	0.114

IVCDs, intraventricular conduction disturbances.

### Prevalence of intraventricular conduction disturbances

The prevalence of IVCDs in the different age groups is shown in Table 2. RBBB (1.58%) was the most prevalent, followed by IRBBB (0.66%) and LAFB (0.66%). ILBBB (0.03%) and LPFB (0.02%) were relatively rare. The prevalence of most IVCD types increased with age, except for ILBBB, NIVCD, and LPFB.

**TABLE 2:** Prevalence of IVCDs by age at baseline.

	Total		<39		40–49		50–59		60–69		≥70		Sex-adjusted *P-*value
							
	*n* = 100,250		*n* = 16,332		*n* = 25,741		*n* = 34,692		*n* = 14,956		*n* = 8,529		
							
	*n*	%	*n*	%	*n*	%	*n*	%	*n*	%	*n*	%	
RBBB	1,583	1.58	61	0.37	203	0.79	451	1.30	434	2.90	434	5.09	<0.001
IRBBB	665	0.66	76	0.47	126	0.49	233	0.67	115	0.77	115	1.35	<0.001
LBBB	93	0.09	4	0.02	2	0.01	23	0.07	28	0.19	36	0.42	<0.001
ILBBB	26	0.03	3	0.02	4	0.02	8	0.02	4	0.03	7	0.08	0.129
NIVCD	155	0.16	37	0.23	47	0.18	49	0.14	17	0.11	5	0.06	0.001
LAFB	661	0.66	37	0.23	88	0.34	217	0.63	165	1.10	154	1.81	<0.001
LPFB	18	0.02	7	0.04	2	0.01	6	0.02	3	0.02	0	0.00	0.164
Total	3,201	3.19	225	1.38	472	1.83	987	2.85	766	5.12	751	8.81	<0.001

IVCDs, intraventricular conduction disturbances; RBBB, right bundle branch block; IRBBB, incomplete RBBB; LBBB, left bundle branch block; ILBBB, incomplete LBBB; NIVCD, non-specific IVCD; LAFB, left anterior fascicular block; LPFB, left posterior fascicular block.

**FIGURE 1:**
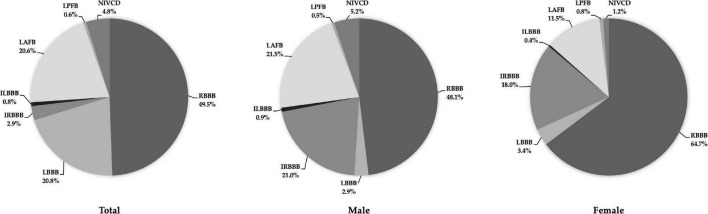
Distribution of prevalent IVCDs in the total study population (***left***), males (***middle***), and females (***right***). Abbreviations as in Table 2.

Males exhibited a higher prevalence of all IVCDs than females (Table 3). For males, the most frequent types of IVCDs were RBBB (48.1%), LAFB (21.5%), and IRBBB (21.0%). For females, the most frequent types of IVCDs were RBBB (64.7%), IRBBB (18.0%), and LAFB (11.5%) (Figure 1).

**TABLE 3:** Prevalence of IVCDs by sex at baseline.

	Total		Male		Female		Age-adjusted *P-*value
				
	*n* = 100,250		*n* = 80,056		*n* = 20,194		
				
	*n*	%	*n*	%	*n*	%	
RBBB	1,583	1.58	1,414	1.77	169	0.84	<0.001
IRBBB	665	0.66	618	0.77	47	0.23	<0.001
LBBB	93	0.09	84	0.11	9	0.05	0.188
ILBBB	26	0.03	25	0.03	1	0.01	0.100
NIVCD	155	0.16	152	0.19	3	0.02	<0.001
LAFB	661	0.66	631	0.79	30	0.15	<0.001
LPFB	18	0.02	16	0.02	2	0.01	0.271
Total	3,201	3.19	2,940	3.67	261	1.29	<0.001

Abbreviations as in Table 2.

### Incidence of intraventricular conduction disturbances

The IPs and IDs of IVCDs in the different age groups are displayed in Table 4. The IP of all IVCDs was 1.70%, while the ID was 3.51 cases per 1,000 person-years. RBBB (0.78%; 1.58 cases per 1,000 person-years), LAFB (0.44%; 0.87 cases per 1,000 person-years) and IRBBB (0.42%; 0.84 cases per 1,000 person-years) were the IVCD types with the highest incidence. The incidence of RBBB, IRBBB, LBBB and LAFB was higher in older groups. ILBBB, NIVCD, and LPFB were rarely detected.

**TABLE 4:** Incidence proportion and incidence density of IVCDs by age after 6 years.

	Total		<39		40–49		50–59		60–69		≥70		Sex-adjusted *P* value
							
	*n* = 97,049		*n* = 16,017		*n* = 25,269		*n* = 33,705		*n* = 14,190		*n* = 7,778		
							
	*n* = 97,049		*n* = 16,017		*n* = 25,269		*n* = 33,705		*n* = 14,190		*n* = 7,778		
	*n*	IP	*n*	IP	*n*	IP	*n*	IP	*n*	IP	*n*	IP	

		ID		ID		ID		ID		ID		ID	
RBBB	757	0.78	48	0.30	134	0.53	280	0.83	174	1.23	121	1.56	<0.001
		1.58		0.55		1.00		1.67		2.65		4.76	
IRBBB	407	0.42	57	0.36	89	0.35	158	0.47	70	0.49	33	0.42	0.023
		0.84		0.66		0.66		0.94		1.04		1.25	
LBBB	44	0.05	1	0.01	3	0.01	13	0.04	14	0.10	13	0.17	<0.001
		0.09		0.01		0.02		0.08		0.21		0.49	
ILBBB	9	0.01	0	0.00	1	0.004	2	0.01	4	0.03	2	0.03	0.311
		0.02		0.00		0.01		0.01		0.06		0.07	
NIVCD	7	0.01	1	0.01	4	0.02	2	0.01	0	0.00	0	0.00	0.924
		0.01		0.01		0.03		0.01		0.00		0.00	
LAFB	423	0.44	34	0.21	90	0.36	154	0.46	96	0.68	49	0.63	<0.001
		0.87		0.39		0.67		0.91		1.43		1.87	
LPFB	7	0.01	2	0.01	1	0.004	4	0.01	0	0.00	0	0.00	0.921
		0.01		0.02		0.01		0.02		0.00		0.00	
Total	1,654	1.70	143	0.89	322	1.27	613	1.82	358	2.52	218	2.80	<0.001
		3.51		1.66		2.43		3.73		5.58		8.92	

IP and ID in the table are described as percentages (%) and cases per 1,000 person-years, respectively. IP, incidence proportion; ID, incidence density; the other abbreviations are as in Table 2.

**FIGURE 2:**
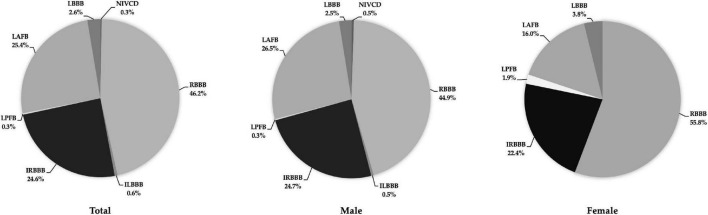
Distribution of incident IVCDs in the total study population (***left***), males (***middle***), and females (***right***). Abbreviations as in Table 2.

Males exhibited a higher incidence than females for all IVCDs, except LPFB (Table 5). For both males and females, the most frequent types of IVCDs were RBBB (44.9% vs. 55.8%, respectively), IRBBB (24.7% vs. 22.4%) and LAFB (26.5% vs. 16.0%) (Figure 2).

**TABLE 5:** Incidence proportion and incidence density of IVCDs by sex after 6 years.

	Total			Male			Female			Age-adjusted *P-*value
				
	*n* = 97,049			*n* = 77,116			*n* = 19,933			
				
	*n*	IP	ID	*n*	IP	ID	*n*	IP	ID	
RBBB	757	0.78	1.58	663	0.86	1.78	94	0.47	0.87	<0.001
IRBBB	407	0.42	0.84	369	0.48	0.98	38	0.19	0.35	<0.001
LBBB	44	0.05	0.09	38	0.05	0.10	6	0.03	0.06	0.629
ILBBB	9	0.01	0.02	9	0.01	0.02	0	0.00	0.00	0.966
NIVCD	7	0.01	0.01	7	0.01	0.02	0	0.00	0.00	0.967
LAFB	423	0.44	0.87	396	0.51	1.05	27	0.14	0.25	<0.001
LPFB	7	0.01	0.01	4	0.005	0.01	3	0.02	0.03	0.225
Total	1,654	1.70	3.51	1,486	1.93	4.08	168	0.84	1.57	<0.001

IP and ID in the table are described as percentages (%) and cases per 1,000 person-years, respectively. IP, incidence proportion; ID, incidence density; the other abbreviations are as in Table 2.

### Association of intraventricular conduction disturbances with cardiovascular diseases

Table 6 shows the relationship between IVCDs and CVDs. IRBBB (OR = 1.17, 95% CI 1.10–1.28), LBBB (OR = 1.35, 95% CI 1.20–1.52) and LAFB (OR = 1.21, 95% CI 1.09–1.34) were significantly associated with HTN. LBBB (OR = 1.12, 95% CI 1.01–1.26) and NIVCD (OR = 1.18, 95% CI 1.01–1.38) were associated with DM. In addition, LBBB (OR = 1.23, 95% CI 1.12–1.36) and LAFB (OR = 1.20, 95% CI 1.10–1.40) were related to prior MI. RBBB, ILBBB, and LPFB were not associated with CVDs.

**TABLE 6:** Association of IVCDs with CVDs.

	RBBB^‡^	IRBBB^‡^	LBBB^†^	ILBBB^†^	LAFB^‡^	LPFB^†^	NIVCD^‡^b
Prior myocardial infarction	0.84 (0.46–1.52)	1.02 (0.58–1.89)	1.23*** (1.12–1.36)	–	1.20*** (1.10–1.40)	1.21 (0.82–1.60)	1.11 (0.69–1.70)
Prior stroke	0.69 (0.43–1.12)	1.24 (0.82–1.90)	0.81 (0.42–1.54)	1.10 (0.74–1.50)	0.92 (0.78–1.22)	–	0.86 (0.60–1.26)
Hypertension	0.93 (0.81–1.08)	1.17*** (1.10–1.28)	1.35*** (1.20–1.52)	1.08 (0.70–1.46)	1.21* (1.09–1.34)	0.94 (0.78–1.21)	0.98 (0.81–1.24)
Diabetes mellitus	0.97 (0.76–1.24)	0.96 (0.74–1.26)	1.12* (1.01–1.26)	0.86 (0.51–1.32)	1.02 (0.64–1.49)	1.08 (0.72–1.50)	1.18* (1.01–1.38)

Numbers in the table are odds ratios (95% confidence intervals). CVD, cardiovascular disease; the other abbreviations are as in Table 2.

^†^Adjusted for age and sex.

^‡^Adjusted for age, sex, body mass index, smoking status, drinking status, antihypertensive medication, antidiabetic medication, and CVDs.

***P < 0.001; *P < 0.05.

## Discussion

### Main findings

To the best of our knowledge, the present study is the first to examine both the prevalence and incidence of all IVCDs in a Chinese community-based population. The prevalence and incidence of most IVCD types increased with age. Males exhibited a higher incidence and prevalence than females for most IVCDs. RBBB, IRBBB, and LAFB were the IVCD types with the highest prevalence and incidence among both males and females.

### Global prevalence of intraventricular conduction disturbances

The prevalence of IVCDs varied in previous studies. In a large French population of 69,186 participants (71.8% males), Monin et al. (6) reported a similar prevalence of IVCDs of 3.93% (vs. 3.67%) for males (aged 32.6 ± 11.3 years) and 0.96% (vs. 1.29%) for females (aged 28 ± 8.3 years). They also classified the type of IVCDs according to the AHA/ACCF/HRS recommendations. The prevalence of IVCDs in the Health 2000 survey of the general population in Finland (51.5% males; 39.5% participants aged ≥ 55 years) was higher than that in our study (9.6% vs. 3.19%, respectively) (5). IVCDs were defined based on Minnesota codes (10), which contains some differences from the AHA/ACCF/HRS recommendations. Moreover, the percentages of cardiovascular comorbidities in the Health 2000 survey were higher than those in the present study, especially among participants with a history of MI (8.9% vs. 2.9%, respectively). In a middle-aged Swiss population (44.8% males; aged 35–75 years), IVCDs were found in one out of 20 participants (5.1%) (11). IVCDs were also classified based on the AHA/ACCF/HRS recommendations. However, that study only included subjects aged over 45, and more than 1/3 of individuals were ≥ 65 years old. In addition, Rasmussen et al. (12) reported an IVCD prevalence of 4.4% in Denmark in a sample of 202,268 individuals (43.3% males). The AHA/ACCF/HRS recommendations were used to define most types of IVCDs. However, the cutoff value for NIVCD was 120 ms in their study (110 ms in the AHA/ACCF/HRS recommendations). In addition, their study excluded participants who were younger than 40 years of age. In the present study, a large proportion of participants (42.0%) were less than 50 years old. Therefore, the variations in prevalence may be due to the specific definitions of the IVCD subtypes used or to the characteristics of the samples included.

Unfortunately, to the best of our knowledge, no investigation concerning the incidence of all IVCDs has been published to date, as some studies investigating the incidence included only certain types of IVCDs.

### Prevalence, incidence, and association with cardiovascular diseases of right bundle branch block and incomplete right bundle branch block

The prevalence of RBBB was 2 times higher among males than among females and was highly age- and sex-dependent in our study. Other studies showed a similar trend (4, 5, 13). The Health 2000 survey demonstrated a similar prevalence of RBBB among both males (1.5%) and females (0.7%) (5). Rasmussen et al. (12) also reported a similar RBBB prevalence of 1.7%. The results from a large French population indicated a lower RBBB prevalence (0.46%) than ours (6). The RBBB prevalence (0.90%) in the Copenhagen City Heart Study was also lower than that in our study (4). In addition, that study reported an incidence of 0.72% among males and 0.30% among females after 5 years (4). This is comparable to the incidence in our study, with a follow-up period of 6 years.

The prevalence of IRBBB in the present study was 0.66%. This was less than that in the Health 2000 survey (1.0%) (5) and in a French population (1.25%) (6). The IRBBB prevalence (3.38% vs. 0.66%) and incidence (1.35% vs. 0.41%) of the Copenhagen City Heart Study were higher than those in our study (4). However, the prevalence of IRBBB in our study was very similar to that in the Copenhagen ECG study (0.65%) (12). Interestingly, Kobza et al. reported a greater IRBBB prevalence of 13.5% among Swiss citizens who underwent conscription for the army (mean age: 19.2 years) (14). Additionally, IRBBB is more common in athletes (median age: 17 years) (15). Thus, it has been suggested that the high prevalence of IRBBB among young individuals may be caused by an enlarged right ventricular cavity and increased cardiac muscle mass, resulting in a conduction delay (16). This is considered a benign phenomenon associated with sport-induced remodeling in athletes.

In the Copenhagen City Heart Study, the risk of RBBB increased with higher systolic blood pressure, whereas IRBBB was not related to any CVD risk factors (4). In the Health 2000 survey, RBBB was related to angina pectoris and peripheral vascular disease, whereas IRBBB was associated with chronic heart failure (5). In the present study, we found that IRBBB was related to HTN and DM. However, RBBB was not associated with any comorbidities in our study. Therefore, the difference in RBBB prevalence among populations may be due to differences in age, sex, and risk factor distribution.

### Prevalence, incidence, and association with cardiovascular diseases of left bundle branch block and incomplete left bundle branch block

LBBB is common in patients hospitalized for heart failure (1) and coronary heart disease (2). The prevalence of LBBB is largely dependent on age and cardiovascular risk profile (5, 17). The present study demonstrated that LBBB, but not ILBBB, was associated with HTN, DM and prior MI. LBBB is rare in young individuals and almost never occurs before 35 years of age, suggesting that it may be an acquired condition (18). Among asymptomatic adults, the estimated prevalence of LBBB ranges between 0.08 and 0.9% (5, 6, 11, 12). The prevalence of LBBB in the current investigation (0.09%) was similar to that in a study by Monin et al. (0.08%) (6) but less than that in studies by Haataja et al. (0.9%) (5), Rasmussen et al. (0.5%) (12), and Bay et al. (0.8%) (11). Taken together, these results exhibit variation in the prevalence of LBBB in different populations. However, the incidence of LBBB in the general population has rarely been investigated and remains unclear. Imanishi et al. (3) observed the incidence of LBBB among 17,361 participants over a 40-year period. The incidence of LBBB was 0.002 cases per 1,000 person-years and 0.018, 0.042, 0.066, and 0.157 for males aged < 50, 50–59, 60–69, 70–79 years, and ≥ 80, respectively. The corresponding incidence rates among females were 0.002, 0.022, 0.036, 0.063, and 0.114. In our study, the incidence increased from 0.01 cases per 1,000 person-years among patients aged < 39–0.49 per 1,000 person-years among those aged > 70, the latter of which was higher than the incidence reported by Imanishi et al. (3).

For ILBBB, the prevalence in the present study (0.03%) was lower than that in the Health 2000 survey (1.0%) (5) but very similar to that in a study by Monin et al. (0.03%) (6). The incidence of ILBBB in our investigation was also very low. We found no other population-based studies on the incidence of ILBBB in our literature search.

### Prevalence, incidence, and association with cardiovascular diseases of left anterior fascicular block and left posterior fascicular block

LAFB is one of the most prevalent types of IVCDs in the general population. The prevalence of LAFB in the present study was lower than that among middle-aged Swiss adults (0.9%) (11), in a large French population (1.1%) (6), and in the general Finnish population (1.0%) (5). Interestingly, Kobza et al. (14) reported an LAFB prevalence of 0.83% among young individuals. LAFB was found in 2.8% of 8,915 presumably healthy individuals engaged in civilian flying activities in Argentina (19). These findings contrast with some previous investigations and the current research. The prevalence was significantly age-dependent in the Health 2000 survey (5) and our study. In addition, LAFB was associated with chronic heart failure in the Health 2000 survey (5) and associated with HTN and prior MI in our study. The differences between studies may be caused by the ethnic diversity and inconsistent distribution of comorbidities between different study samples.

LPFB is extremely rare because the posterior division of the left bundle branch is the least vulnerable segment of the intraventricular conduction system (20). The prevalence of LPHB in the present study was only 0.02%. After 6 years of follow-up, the incidence was as low as 0.01%. No comorbidity was associated with LPHB in our study. The prevalence of LPHB in the Health 2000 survey was greater (0.1%) than our own, but, similar to our findings, was not related to any major CVDs (5).

### Prevalence, incidence, and association with cardiovascular diseases of non-specific intraventricular conduction disturbance

Epidemiological data on NIVCD, especially its incidence, are rarely reported. In the present study, the prevalence in the general population was 0.15%, and individuals with NIVCD were preponderantly male. Only 7 participants developed NIVCD during the 6-year follow-up. The prevalence in a French population was lower than that in our study (0.05% vs. 0.15%) (6). The prevalence of NIVCD in the Copenhagen ECG study was as high as 1.5% of the study population (12). In the Health 2000 survey, the prevalence of NIVCD was 0.6% (5); additionally, NIVCD was associated with MI, angina pectoris, chronic heart failure, and stroke. However, we only found an association between NIVCD and DM. Therefore, the differences in prevalence may be attributed to demographic characteristics and dissimilar distributions of associated pathologies.

### Limitations

The present study has some potential limitations. First, the Kailuan Study was based on workers from the Kailuan Company, most of whom were coal miners. Therefore, female enrollment was lower than that of males, with a male-to-female ratio of approximately 4:1. Second, echocardiography examinations to assess cardiac function for congestive heart failure were not performed. In addition, access to national registries was limited. Therefore, the association between IVCDs and some other CVDs (e.g., heart failure, pulmonary diseases, peripheral vascular disease, and chronic coronary artery disease) could not be investigated. Finally, individuals aged < 30 or > 80 accounted for a minority of the study population. Hence, the generalizability of conclusions regarding the prevalence and incidence of IVCDs in the global population may depend on age group.

## Conclusion

The prevalence and incidence of all IVCDs in the study population were 3.19% and 1.70% (3.51 cases per 1,000 person-years), respectively. IVCDs differed in prevalence and incidence according to sex and age; they were more frequent among males and older individuals. RBBB, IRBBB, and LAFB were the IVCD types with the highest prevalence and incidence. IRBBB, LBBB, LAFB, and NIVCD showed disparate associations with other CVDs, whereas RBBB, ILBBB, and LPFB did not.

## Data availability statement

The raw data supporting the conclusions of this article will be made available by the authors, without undue reservation.

## Ethics statement

The studies involving human participants were reviewed and approved by Ethics Committee of Kailuan General Hospital. The patients/participants provided their written informed consent to participate in this study.

## Author contributions

BW, ZW, SW, and YX designed the study. BW, ZW, XY, XH, and YY collected the patient data, edited the images, and performed the statistical analysis. BW and ZW drafted the manuscript. XY, XH, YY, HC, SW, and YX critically revised the manuscript and approved the article. All authors contributed to the article and approved the submitted version.
